# Navigating the kidney disease care cascade in type 2 diabetes: insights from the national diabetes registry

**DOI:** 10.1186/s12889-026-28520-9

**Published:** 2026-07-27

**Authors:** Soong Yiing Yap, Shamsul Azhar Shah, Ruslinda Mustafar, Noraryana Hassan

**Affiliations:** 1 Department of Public Health Medicine, Faculty of Medicine, National University of Malaysia, Jalan Yaacob Latif Kuala Lumpur, Bandar Tun Razak, Cheras, Wilayah Persekutuan Kuala Lumpur, 56000 Malaysia; 2 Department of Medicine, Faculty of Medicine, National University of Malaysia, Jalan Yaacob Latif Kuala Lumpur, Bandar Tun Razak, Cheras, Wilayah Persekutuan Kuala Lumpur, 56000 Malaysia; 3 Disease Control Division, Federal Government Administration Centre, Ministry of Health Malaysia, Putrajaya, 62590 Malaysia

**Keywords:** Diabetic kidney disease, Unmet needs, Diabetes, Care cascade, Malaysia

## Abstract

**Background:**

Diabetic kidney disease (DKD) is a common complication among people with type 2 diabetes (T2D) and is a leading cause of chronic kidney disease (CKD). Malaysia ranks among the highest diabetes-related CKD rates worldwide, and despite research emphasis on diabetes and its complications, the unmet needs for DKD care remain largely unexplored. This study aimed to identify the unmet need for diagnosis, treatment, and control of DKD using the care cascade among patients with T2D receiving follow-up care in government health clinics in Malaysia.

**Methods:**

A cross-sectional analysis of the National Diabetes Registry (NDR) 2023 was conducted. Inclusion criteria were adult patients with T2D that received DKD screening or had a diagnosis of nephropathy. DKD was defined as either documented nephropathy in the NDR, or abnormal estimated glomerular filtration rate (eGFR) or albuminuria. Treatment was defined as the documented prescription of either angiotensin converting enzyme inhibitor (ACEi)/angiotensin-receptor blocker (ARB)/Sodium-glucose Cotransporter 2 inhibitors (SGLT2i)/ Glucagon-like peptide-1 receptor agonist (GLP1-RA) and statin, whereas control was defined as achieving glycosylated haemoglobin A1c (HbA1c) and blood pressure targets. Multivariable logistic regression was done to identify the determinants for unmet need at each stage.

**Results:**

The prevalence of DKD among diabetic patients was 53.2% (56949/107099). The prevalence of total unmet need for care was 97.6%, with deficiencies observed across all stages; diagnosis (74.6%), treatment (27.9%) and control (86.8%). The two crucial gaps identified in this care cascade were for uncontrolled and undiagnosed DKD, with uncontrolled blood pressure identified as the main contributor for uncontrolled DKD. Risk factors for unmet need were female sex (adjusted odds ratio (AOR) 1.23–1.39), middle age (AOR 1.38–3.15), and hyperlipidaemia (AOR 1.10–1.52).

**Conclusions:**

The DKD care cascade identified a notable gap in meeting care needs, with a consistent deficiency in care at each stage. This study findings serve as a benchmark and framework for the evaluation and improvement of DKD care in Malaysia. While addressing the factors associated with undiagnosed and uncontrolled DKD are crucial, further evaluation of home-based blood pressure should be done prior to intensifying control measures.

**Supplementary Information:**

The online version contains supplementary material available at https://doi.org/10.1186/s12889-026-28520-9.

## Introduction

Type 2 diabetes (T2D) is a highly prevalent disease which affects an estimated 422 million people globally, and these numbers are expected to further increase [1]. Diabetic kidney disease (DKD) is one of the most common complications noted among patients with T2D [2], with reported estimates ranging from 26.7% to 83.7% [3]. The development of kidney damage is a strong independent risk factor for the development of cardiovascular events and mortality [4]. Unmet need has been defined as the gap between received health care and appropriate services [5], and as such has been used to indicate the capacity of health systems [6]. Stokes et al. has since defined unmet need quantitatively as the summation of the subcategories with deficiencies in the care cascade [7]. 

Malaysia has the highest proportion of chronic kidney disease (CKD) attributable to diabetes in the world [8]. In view of the health and economic burdens of this disease [9, 10], strict surveillance of patients with T2D is required for the early diagnosis, treatment, and control of DKD. This forms the care cascade, which is an effective graphical portrayal of the consecutive steps for the control of non-communicable diseases (NCD) [7, 11–15]. 

We have yet to come across a published report on the DKD care cascade in Malaysia. As the Malaysian National Diabetes Registry (NDR) was established to monitor patient outcomes and support research to improve quality of care, this study aimed to develop a service-based DKD care cascade to benchmark the performance of the public primary-care system in DKD diagnosis, treatment, and control among patients captured in the registry.

## Methodology

### Data source

A cross-sectional study was done using the NDR Clinical Audit Dataset. The NDR is a national database which collects information annually on T2D patients attending government clinics which were selected via simple random sampling. Data on patients with T2D attending follow-up in government health clinics between 1st January 2023 to 31st August 2023 in the NDR, was extracted on 17th November 2023. The study was proposed after the consolidation of the 2023 NDR to prevent bias in selection and collection of data.

The sample size was calculated using the equation n = *Z*^2^[(*P*(1 − *P*)/*e*^2^)] [16], where *Z* is the confidence level, *P* is the prevalence of unmet need and *e* is the error margin. Using *Z* = 1.96, *e* = 0.05, *P* = 0.25 (estimated undiagnosed DKD) [17], 0.68 (estimated untreated DKD) [18], and 0.85 (estimated uncontrolled DKD) [19]. The minimum sample size required for each stage of the care cascade respectively were 291, 334, and 193 participants.

### Study population

The inclusion criteria of this study were adult patients with T2D who had received DKD screening or had the diagnosis of nephropathy in the NDR 2023 dataset. DKD screening was defined as either a result was present for either serum creatinine or urine screening based on the recommendations for DKD diagnosis from our local clinical practice guideline (CPG) [19]. Urine screening was defined as whether a result was present for either urine microalbumin or urine protein test, whereby a positive result would likely indicate the presence of albuminuria [19]. Patients without documented nephropathy diagnosis in the NDR and missing either creatinine or urine results had to be excluded as their kidney function could not be assessed in this study. All participants which fulfilled study criteria were included in the analysis.

**Fig. 1:**
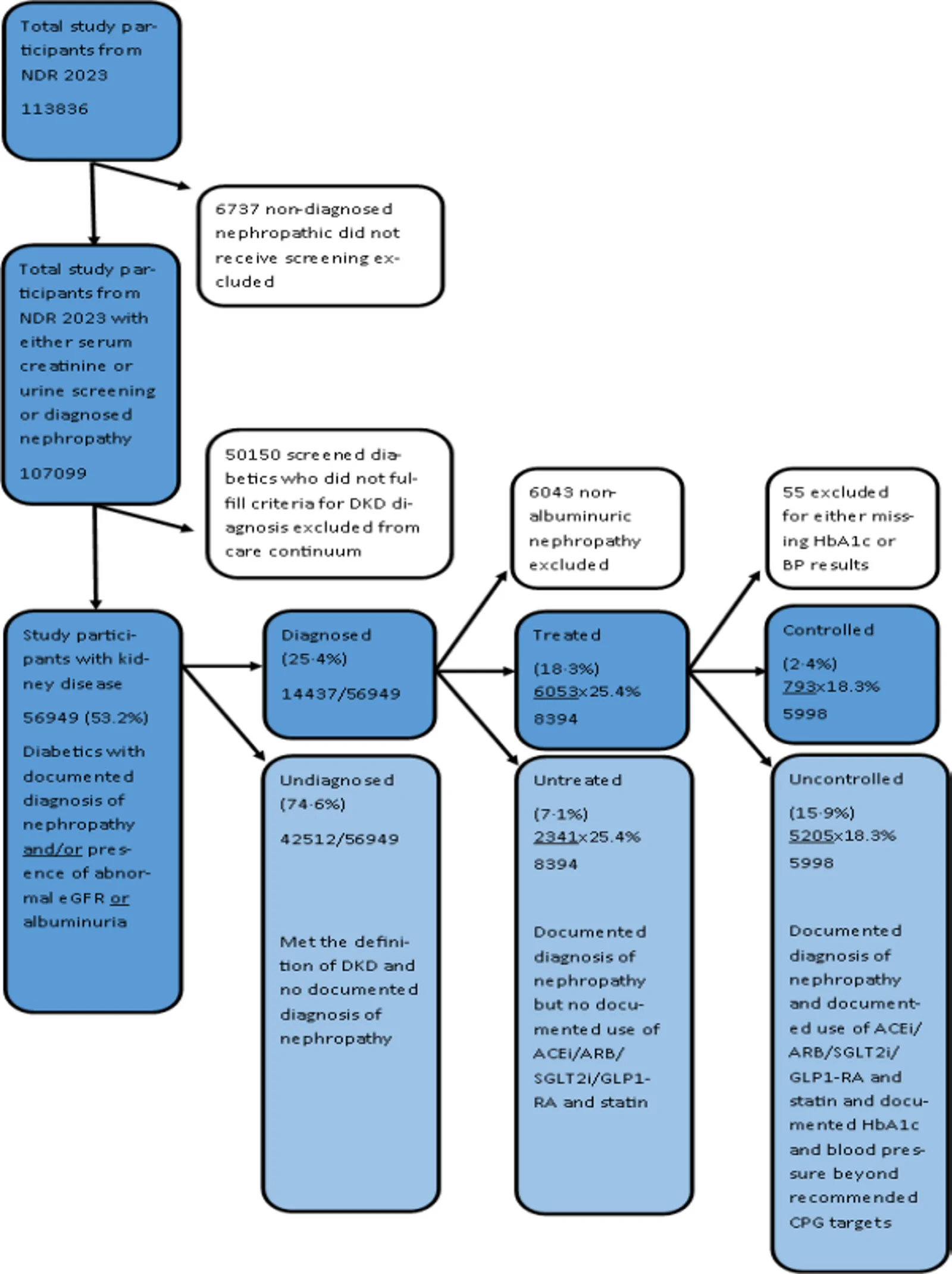
Classification of patients across the DKD care cascade Out of 113,836 patients in the NDR, 6737 did not have DKD screening results and 50,150 with screening results which did not fulfil criteria for DKD diagnosis were excluded. The remaining 56,949 patients were used to construct the DKD cascade, out of which 25.4% had diagnosed disease. For the next stage of the care cascade, 6043 non-albuminuria nephropathy cases were excluded. A total of 18.3% of diagnosed DKD patients received treatment recommended by CPG. For the final stage of the care cascade, 55 cases had to be excluded as HbA1c or blood pressure information were missing. Only 2.4% of the DKD population had controlled disease

Underlying DKD was defined as patients with T2D with either documented nephropathy diagnosis, or the presence of abnormal estimated glomerular filtration rate (eGFR) or albuminuria for those without documented nephropathy diagnosis in the NDR [19]. The criteria for each stage in the DKD care cascade were shown in Fig. 1. The study population were categorized into four mutually exclusive groups which were the endpoints of this study: 1)undiagnosed (no documented diagnosis of nephropathy in the NDR with either abnormal eGFR or albuminuria); 2)diagnosed, untreated (documented diagnosis of nephropathy in the NDR with albuminuria, but no documented angiotensin converting enzyme inhibitor (ACEi)/angiotensin-receptor blocker (ARB)/Sodium-glucose Cotransporter 2 inhibitors (SGLT2i)/ Glucagon-like peptide-1 receptor agonist (GLP1-RA) and statin treatment);3)treated, uncontrolled (documented diagnosis of nephropathy with albuminuria on ACEi/ARB/SGLT2i/GLP1-RA and statins, but unable to achieve both HbA1c and blood pressure control); and 4)treated, controlled (documented diagnosis of nephropathy with albuminuria on ACEi/ARB/SGLT2i/GLP1-RA and statins with good control of HbA1c and blood pressure). Assessment of DKD treatment was derived from CPG recommendations that patients with DKD and albuminuria should be treated with either ACEi/ARB/SGLT2i/GLP1-RA, with additional recommendations that statins be prescribed for primary and secondary prevention of cardiovascular disease [19]. 

eGFR was calculated using the 2009 CKD-epidemiology formula as recommended by the Malaysian CPG [19]. For this study, abnormal eGFR was defined as < 60 ml/min/1.73m^2^ for those 70 years and below [19], or a lower cut point of < 45 ml/min/1.73m^2^ for those who were older than 70 years due to physiological decline of eGFR with age [20]. Glycaemia and blood pressure targets were HbA1c < 7.0%, systolic blood pressures between 120 and 130 mmHg [19, 21], and diastolic blood pressure between 70 and 80 mmHg for those less than 60 years old. For those 60 years and above, more lenient targets were set to reduce the risk of hypoglycaemia and hypotension which was defined as HbA1c level < 8.0%, systolic blood pressure between 120 and 139 mmHg, and diastolic blood pressure between 70 and 89 mmHg [19]. The flowchart for the DKD care cascade was summarised in Fig. 1.

### Variables

The demographic details of patients that were included in this study were age, sex, and ethnicity. Other characteristics of patients included in this study were smoking status, duration of diabetes, presence of comorbidities (hypertension/dyslipidaemia), body mass index (BMI), and presence of diabetic complications (retinopathy, ischemic heart disease (IHD), cerebrovascular disease (CeVD), diabetic foot ulcer (DFU), and amputation). The classification of BMI was done according to The 2023 Malaysian Obesity CPG categories of underweight (< 18.5 kg/m^2^), normal (18.5–22.9 kg/m^2^), overweight (23.0–27.4 kg/m^2^), Obese I (27.5–32.4 kg/m^2^), Obese II (32.5–37.4 kg/m^2^), and Obese III (≥ 37.5 kg/m^2^) [22]. The presence of comorbidities and complications were based on clinical diagnoses. For serum cholesterol, the cut points were derived from CPG definitions for controlled triglyceride (≤ 1.7 mmol/L), controlled high-density lipoproteins (HDL) (male: >1.0 mmol/L, female: >1.2 mmol/L) and controlled low-density lipoproteins (LDL) (≤ 2.6 mmol/L) [19]. 

This study was conducted in accordance with the Medical Review and Ethics Committee (MREC), Ministry of Health, Malaysia guidelines. MREC, Malaysian Director-General of Health and NCD department approval and registration of this study in the National Medical Research Registry of Malaysia (NMRR ID-23-02495-35 S) were done prior to obtaining the NDR 2023 dataset. Requirement for informed consent to participate was waived by MREC due to the use of anonymized secondary data for this study. All personal identifiers were removed from dataset to ensure anonymity of patients. Preparation of this research was done using the Strengthening the Reporting of Observational studies in Epidemiology (STROBE) guidelines for cross-sectional studies.

### Statistical analysis

Descriptive statistics were used to calculate the prevalences and the proportions of the care cascade. Mean and standard deviation were used to describe normally distributed quantitative variables. Acceptable range for normality was skewness ranging from − 1 to + 1 [23] and kurtosis ranging from − 3 to + 3 [24]. Independent t-test were used to analyse the differences between normally distributed quantitative variables. Quantitative variables which had a skewed distribution were described as median and interquartile range, and Mann-Whitney U test was done to analyse the differences between groups. Qualitative variables were analysed using Pearson’s chi-square test. Sensitivity analysis were done for entire dataset using different systolic blood pressure cutoff values of < 120mmHg [25], <140mmHg and < 150mmHg [19]. 

As each stage of the care cascade had a binary outcome, logistic regression was used to identify the factors predisposing the patient to the unmet need for DKD care. Independent variables with p-value < 0.25 using univariable logistic regression were further analysed using multivariable logistic regression analysis using enter method [26]. The final logistic regression models were developed using the purposeful model-building approach described by Hosmer et al., including assessment of interaction and confounding among the independent variables [27]. A p-value of less than 0.05 was taken to be statistically significant. Analyses were done using IBM Statistical Package for Social Sciences Version 20.

## Results

There were 113,836 patients in the NDR 2023, whereby the mean age was 61.9 (11.4) years. Majority were female (*n =* 70525; 62.0%), of Malay ethnicity (*n =* 72514; 63.7%), and were overweight (*n =* 96026; 84.4%). The mean years of being diagnosed with diabetes was 8.3 (6.1) years.

A total of 6737 patients without nephropathy diagnosis who did not receive either serum creatinine or urine screening had to excluded from the dataset as the status of their kidney function could not be ascertained. The characteristics of the patients with T2D without kidney disease and those with underlying nephropathy were shown in Table 1. Missing values were noted in the dataset, ranging from 3.4% to 19.0%. Exploration of missing values was done; it was found that the elderly and those with comorbidities were less likely to have missing values. List-wise deletion was done for variables with missing data.

**Table 1:** Categorical and continuous variables of 2023 National Diabetes Registry patients

Correlate			Without DKD (*n* = 50150)		With DKD (*n* = 56949)		*p*-value
			*n*	%	*n*	%	
Gender							
Male			17,477	34.85	23,033	40.44	< 0.001
Female			32,673	65.15	33,916	59.56	
Ethnicity							< 0.001
Malay			31,341	62.49	38,025	66.77	
Chinese			7523	15.00	7833	13.75	
Indian			3867	7.71	3540	6.22	
Non-malay native			6502	12.97	6484	11.39	
Others			917	1.83	1067	1.87	
Concomitant comorbidities							
Presence of Hypertension			38,578	76.93	48,777	85.66	< 0.001
Presence of Dyslipidaemia			38,412	76.60	46,479	81.63	< 0.001
Smoking status							< 0.001
Yes			2531	5.05	3284	5.77	
No/not known			47,619	94.95	53,665	94.23	
Presence of diabetes-related complication							
Retinopathy			4150	8.28	8082	14.19	< 0.001
Ischaemic heart disease			2006	4.00	3548	6.23	< 0.001
Cerebrovascular disease			702	1.40	1484	2.61	< 0.001
Diabetic foot ulcer			271	0.54	819	1.44	< 0.001
Amputation			140	0.28	521	0.91	< 0.001
			Mean (SD)		Mean (SD)		
Age, years			60.67 (11.31)		62.93 (11.10)		< 0.001
Duration of diabetes, years			7.25 (5.54)		9.17 (6.31)		< 0.001
Body Mass Index^a^, kg/m^2^			27.19 (6.61)		27.47 (6.78)		< 0.001
HbA1c^a^, %			6.90 (2.10)		7.30 (2.70)		< 0.001
Systolic blood pressure, mmHg			134.35 (14.70)		136.52 (15.87)		< 0.001
Diastolic blood pressure, mmHg			77.32 (9.85)		76.58 (10.41)		< 0.001
Triglyceride^a^, mmol/L			1.30 (0.80)		1.40 (0.90)		< 0.001
HDL-cholesterol^a^, mmol/L			1.30 (0.42)		1.26 (0.43)		< 0.001
LDL-cholesterol^a^, mmol/L			2.60 (1.30)		2.60 (1.40)		0.880

*DKD *Diabetic kidney disease,* SD* Standard deviation, *HbA1c* Glycosylated haemoglobin A1c, *HDL* High-density lipoprotein, *LDL* Low-density lipoprotein

^a^Median (interquartile range)

Overall prevalence of DKD in this population was 53.2% (56949/107099). The DKD population had a higher likelihood of being male, older, have longer duration of T2D, have comorbidities, smoke and have other diabetic complications. They were also prone to have higher BMI, HbA1c, systolic blood pressure and triglyceride with a lower diastolic blood pressure and HDL levels when compared to the patients with T2D without DKD.

### Diabetic kidney disease care cascade

Patients without nephropathy diagnosis and had normal DKD screening results in the NDR were excluded, leaving a total of 56,949 patients to construct the care cascade. Total positive urine screening tests for either urine microalbumin or urine protein was 44,558 while 29,111 were noted to have abnormal eGFR. Adjustments in the calculation for unmet need were done at each stage of the care cascade to account for the number of excluded cases as shown in Fig. 1.

Patients were categorized to each care cascade group based on their characteristics shown in Fig. 1. We noted 25.4% (*n* = 14437) of the DKD population had a recorded diagnosis of nephropathy in the NDR. We followed CPG recommendations for diabetic nephropathy patients with albuminuria to receive ACEi/ARB/SGLT2i/GLP1-RA and statin, hence the next stage was constructed to cater to the 8394 patients with diagnosed nephropathy and albuminuria. There was no missing information regarding treatment received, whereby 5094 patients received ACEi (60.7%), 1427 (17.0%) received ARB, 116 patients received SGLT2i (1.4%) and one patient received GLP1-RA. For statins, it was noted that 7765 (92.5%) of those with nephropathy diagnosis received this medication. This translated to a total of 6053 patients receiving treatment as recommended by the CPG.

We conducted an exploratory analysis to evaluate potential discrepancies between documentation and treatment among patients classified as having undiagnosed DKD. Among 42,512 patients without a recorded DKD diagnosis, 85.1% (36164) had albuminuria. A total of 1225 patients remained after excluding patients with conditions that may independently justify ACEi/ARB/statin prescription such as documented hypertension, IHD, dyslipidaemia, or CeVD. These findings suggest that approximately 3% of patients labelled “undiagnosed” may have had partial clinical recognition of renal risk, but did not have receive proper documentation.

To assess for control of DKD, entries with either missing Hba1c, systolic or diastolic blood pressure were excluded, leaving 5998 entries to be evaluated. A total of 3153 (52.6%) patients were able to achieve their HbA1c targets, however only 1388 (23.1%) patients were within their blood pressure targets. Out of the 4610 patients who were unable to achieve their blood pressure targets, we found that most patients struggled with high systolic blood pressure. Sensitivity analysis showed that the percentage of patients achieving systolic blood pressures of < 120mmHg, < 140mmHg, and < 150mmHg were 11.4%, 57.2% and 78.8% respectively. Only 793 (13.2%) of the DKD population were able to achieve both glycaemia and blood pressure controls recommended by the CPG.

### Prevalence of unmet needs according to care cascade stage

**Fig. 2:**
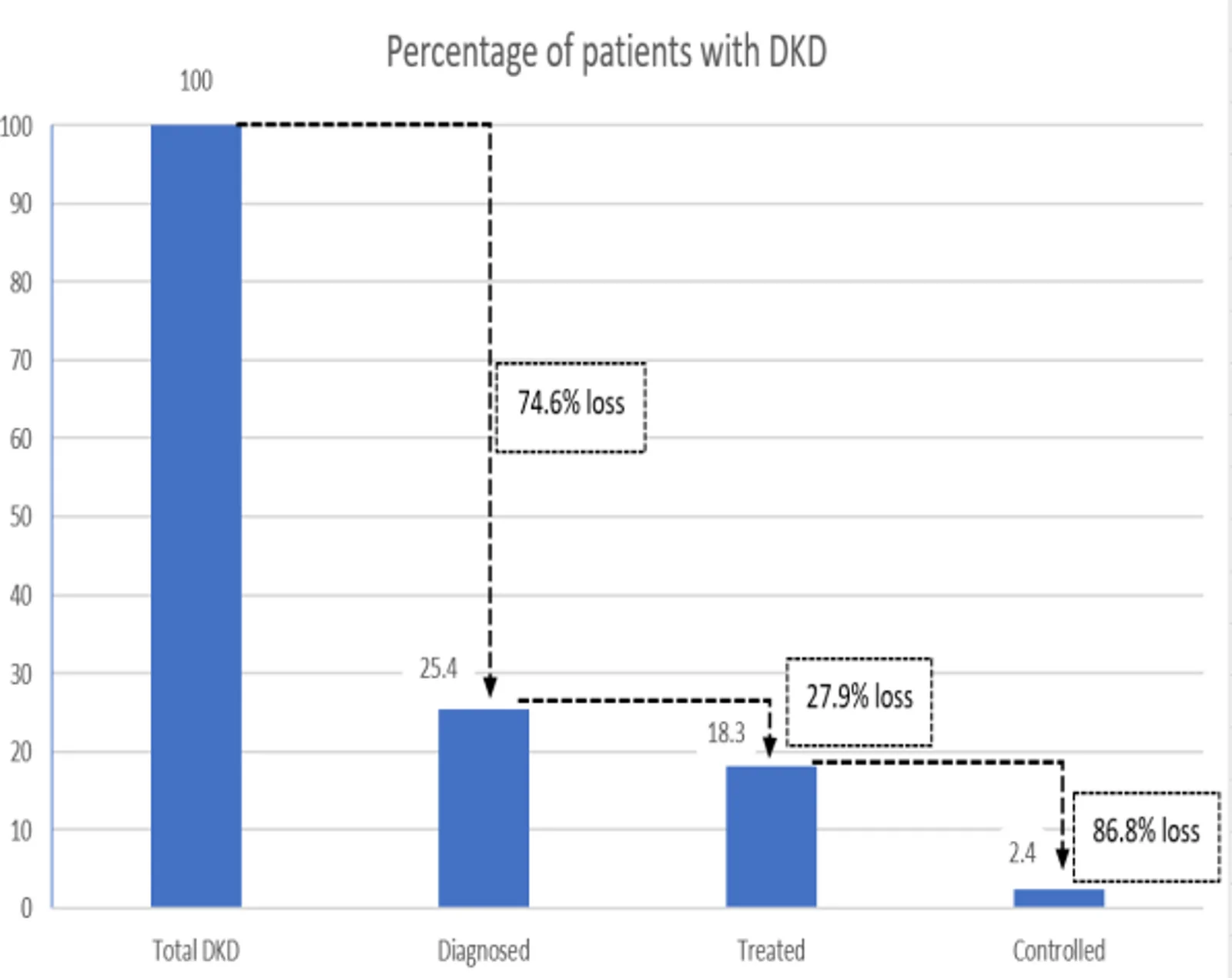
Unmet DKD needs from loss to care at each stage of the DKD care cascade Of those with DKD, 25.4% received a diagnosis of DKD, representing a 74.6% loss to diagnosis. Of those who had been diagnosed, 72.1% received treatment as recommended by clinical practice guidelines, which translates to a loss of 29.9% from treatment. Of those who received recommended treatment, 13.2% had controlled HbA1c and blood pressure, representing a 86.8% loss due to uncontrolled disease

The unmet needs across the DKD care cascade were shown in Fig. 2. There was a loss of 74.6% of patients for DKD diagnosis, followed by further loss of 27.9% who did not receive treatment in the second stage of the care cascade. For the final stage for disease control, another 86.8% had the unmet need for glycaemia and blood pressure control. Overall, this translated to 97.6% of the DKD population having unmet needs across the care cascade, with the largest deficiency caused by uncontrolled DKD. Sensitivity analysis revealed total unmet need calculations range from 92.3% to 97.9% using the different systolic blood pressure cut points stated earlier.

### Predictors of DKD unmet needs

The results of the multivariable analyses for undiagnosed, untreated, and uncontrolled DKD are presented in Supplementary Table 1. DKD stage was excluded from the logistic regression models due to significant interaction effects with several independent variables, which would have required the removal of essential covariates and reduced model interpretability. To support this modelling decision, additional descriptive analyses stratified by DKD stage are presented in Supplementary Table 2. These distributions show that undiagnosed and uncontrolled cases are more concentrated in the earlier stages, whereas untreated cases occur across the full spectrum of disease.

Being female, younger age, of minority ethnicity, having higher diastolic blood pressure and LDL levels were found to be risk factors for undiagnosed DKD, whereas the presence of hypertension, lower diastolic blood pressure and uncontrolled HDL had higher likelihood to have diagnosed DKD.

Patients younger than 40 years old, of other ethnicities, history of amputation, lower systolic blood pressure, and uncontrolled LDL were at higher risk to not receive treatment according to CPG. Whereas those between 40 and 79 years old, of Chinese ethnicity and had higher BMI were noted to have higher chances of having treated DKD as shown in Supplementary Table 1.

HbA1c and blood pressure were omitted from the analysis for uncontrolled DKD as these variables were the determinants for DKD control. Similar to untreated DKD, being female, between 40 and 59 years old, and had uncontrolled LDL had higher risk of uncontrolled disease.

## Discussion

From our findings, we noted that 53.2% of patients with T2D attending government health clinics had underlying nephropathy. This percentage was somewhat similar to the prevalences reported locally and abroad [3, 9, 28]. We reported the first national DKD unmet needs from the care cascade. The largest hurdle for disease control was also noted in the care cascades for other non-communicable diseases such as T2D [7, 12] and hypertension [13, 15]. While intriguing that our study reported a much higher unmet need in comparison to other care cascades, this finding was not surprising as we incorporated both glycaemia and blood pressure targets whereas other studies focused on a single target.

Our study reported similar glycaemia and blood pressure controls as other DKD populations [29, 30], whereby emphasis needs to be placed on blood pressure control which was below the national target of 60% [8]. Sensitivity analysis showed that uncontrolled systolic blood pressure, which were beyond both CPG and Kidney Disease: Improving Global Outcomes (KDIGO) targets, was the main obstacle in achieving DKD control. Stringent systolic blood pressure targets according to KDIGO guidelines was to reduce the risk of cardiovascular events, thus improving morbidity and mortality rates among DKD populations [25]. However, there was discrepancy in the methods of measuring blood pressure: KDIGO recommended standardized office blood pressure measurements, whereas routine office blood pressure was used in this study which could result in higher blood pressure measurements due to “white coat” syndrome [25]. Despite this discrepancy, difficulties in achieving blood pressure targets were also noted in Italy among patients with T2D with albuminuria, whereby only 13.3% were within targets [31]. 

In our study, the second largest gap was for the diagnosis of DKD, which posed a considerable unmet need for other NCD care cascades [7, 11–14, 28]. An estimated 50% of people with T2D have undiagnosed DKD despite undergoing screening tests for kidney disease [32, 33], whereby another half of them had already progressed to late-stage DKD [34]. Missed diagnosis of DKD is not uncommon, with an average lag of two years for healthcare providers to diagnose DKD after the emergence of laboratory evidence for DKD has been reported [33]. Several factors could be attributed to the high rates of undiagnosed DKD. As patients with early DKD are mainly asymptomatic [34], they may refuse repeating the tests to confirm the diagnosis [35]. From the healthcare provider’s perspective, diagnosing elderly patients with DKD poses a challenge as there was no recommended eGFR cut-point in the CPG for those aged 70 years and above due to physiological decline in eGFR [19]. Healthcare providers may also miss diagnosing DKD among patients under regular follow up due to misperception that late-stage DKD is only diagnosed among new patients with T2D, those who have defaulted, those with poor adherence, or those with uncontrolled comorbidities [36]. For early stage DKD, some providers did not think the disease would progress and were unsure of treatment options [36]. Recommendations to bridge this gap are to increase healthcare provider awareness and to create a user-friendly system in the electronic health records which can flag abnormal DKD screening results, which could be reviewed during patient’s follow up to alert the provider about a possible DKD diagnosis [37]. 

Our study noted that 79.1% of diagnosed patients with DKD received treatment, which was within our national goal [8]. While our rates of SGLT2i usage is less than the reported 6% by the DKD population in Korea, both studies reveal the staple treatment for DKD care consisted of renin-angiotensin system blockers [29]. Increasing the rates of SGLT2i usage could potentially reduce the unmet need for both glycaemia and blood pressure control [19, 21, 25, 34], however this treatment should be prescribed with caution for those with advanced DKD [19, 29]. Discontinuation of ACEi due to side effects of this medication and worsening creatinine profile may also contribute to this gap in the care cascade [19]. 

We also noted that female patients were consistently at higher risk for unmet need. There might be gender- and sex- linked characteristic influencing unmet needs [38], as women were also found to be more prone for kidney function decline and failure [39–41]. This phenomenon could be due to poorer clinical controls noted among female patients with T2D [41, 42]. Our study draws similarities with other studies which highlight that the younger patient population with T2D has higher unmet need for disease control [43]. Hence, while increasing age has been found to be a consistent independent risk factor for the development of DKD among T2D; [29, 44] the reason that younger DKD populations have higher unmet need could be due to their internal struggle to cope with competing needs and responsibilities [45, 46]. The presence of aggressive young onset T2D, for which being female is a risk factor, could also explain these factors for unmet need [47]. It was interesting to note that factors for unmet need also differ with the usual risk factors identified for DKD [28, 48], which may in turn prove to be protective against unmet need as supported by the reduced likelihood of missing values for this sub-population noted in this study.

Other studies have also noted differing unmet needs across ethnicities, whereby Chinese patients with T2D was also found to be more likely to have treated and controlled diabetes, while Malay and Indian patients were at higher risk of being unaware of their diagnosis and not receiving treatment [49, 50]. This finding could be explained by the stipulated differences in genetics, environment and culture between ethnicities [51]. Hyperlipidaemia was found to be a risk factor for uncontrolled DKD, while high HDL levels increased the chances of having diagnosed DKD in this study. Interestingly, similar associations between serum cholesterol and DKD have also been shown in other studies, notably lower triglyceride levels and high HDL levels were found to be protective against DKD [28, 52]. These findings could be explained by the plausible role of hyperlipidaemia in the pathogenesis of DKD [53]. 

### Strengths and limitations

While some data were missing at random, list-wise deletion was applied and resulted in findings that are valid and comparable with other studies [29, 31]. The cross-sectional design precludes causal inference between predictors and outcomes. Other determinants of health and long-term treatment adherence, which may influence unmet needs, were not captured, as the analysis was limited to variables available in the NDR [11]. Measurements in the NDR reflect real-world clinical practice but were not standardized across facilities.

A proportion of undiagnosed patients may not have been captured; however, the high DKD screening rate in our sample (94.1%) minimizes this limitation and strengthens the validity of our findings. Our exploratory analysis further indicates that only a small subset (3%) of patients classified as undiagnosed DKD may have received treatment without documentation, supporting that the majority of the undiagnosed population was correctly identified. DKD prevalence may still be overestimated, as changes in nephropathy status could not be tracked and DKD was defined by a single abnormal urine test or eGFR. To mitigate this, we applied a lower eGFR cutoff for the elderly, consistent with Delanaye et al., [20] and our approach aligns with other published studies [48]. 

Despite these limitations, this study included a large nationwide sample and, through the care cascade framework, highlights gaps in DKD management in Malaysian government health clinics, providing actionable insights for health-system improvement.

## Conclusion

This study shows there was a significant unmet need for DKD care in government health clinics in Malaysia, especially for the control and diagnosis of disease. Furthermore, our findings recommend that focus for DKD care need to be shifted to patients who are female, age less than 60 years old, and those with hyperlipidaemia. While our study serves as a benchmark and framework for evaluation of DKD care, evaluation of home-based blood pressure measurements of patients is needed prior to intensifying control measures.

## Supplementary Information

Supplementary Material 1.

Supplementary Material 2.

## Data Availability

Data from the NDR are available upon obtaining written permission from the Malaysian Director-General of Health.

## Abbreviations

ACEi: Angiotensin converting enzyme inhibitor
AOR: Adjusted odds ratio
ARB: Angiotensin-receptor blocker
CKD: Chronic kidney disease
CPG: Clinical practice guideline
DKD: Diabetic kidney disease
eGFR: Estimated glomerular filtration rate
GLP1-RA: Glucagon-like peptide-1 receptor agonist
HbA1c: Glycosylated haemoglobin A1c
NDR: National Diabetes Registry
SGLT2i: Sodium-glucose Cotransporter 2 inhibitors
T2D: Type 2 diabetes
