# Supplementary material for: Navigating the kidney disease care cascade in type 2 diabetes: insights from the national diabetes registry
Source: BMC Public Health. 2026 Jul 27;26:3035. doi: 10.1186/s12889-026-28520-9 (PMC13689289; doi:10.1186/s12889-026-28520-9)
Supplement: Supplementary file 1 [file 12889_2026_28520_MOESM1_ESM.docx]

**Supplementary Table 1: Predictors of Diabetic Kidney Disease unmet needs**

| Characteristics | | | Diabetic Kidney Disease | | | | | | | | | | | | | | | | |
| --- | --- | --- | --- | --- | --- | --- | --- | --- | --- | --- | --- | --- | --- | --- | --- | --- | --- | --- | --- |
|  |  |  | Undiagnosed (*n*=42512) | | | | |  | Untreated (n=2363) | | | | |  | Uncontrolled (n=5185) | | | | |
|  |  |  | AOR | 95% confidence interval | | | *p*-value |  | AOR | 95% confidence interval | | | *p*-value |  | AOR | 95% confidence interval | | | *p*-value |
| Sex | | |  |  |  |  | <0.001 |  | NA |  |  |  |  |  |  |  |  |  |  |
|  | Male | | Ref. |  |  |  |  |  |  |  |  |  |  |  | Ref. |  |  |  | <0.001 |
|  | Female | | 1.26 | 1.21 | - | 1.32 |  |  |  |  |  |  |  |  | 1.51 | 1.29 | - | 1.77 |  |
| Age, years | | |  |  |  |  | <0.001 |  |  |  |  |  | <0.001 |  |  |  |  |  | <0.001 |
|  | < 40 | | 2.75 | 2.26 | - | 3.35 | <0.001 |  | 1.70 | 1.10 | - | 2.63 | 0.017 |  | 2.55 | 0.76 | - | 8.56 | 0.131 |
|  | 40-59 | | 1.89 | 1.70 | - | 2.09 | <0.001 |  | 0.69 | 0.54 | - | 0.88 | 0.002 |  | 2.93 | 1.90 | - | 4.51 | <0.001 |
|  | 60-79 | | 1.35 | 1.23 | - | 1.48 | <0.001 |  | 0.71 | 0.57 | - | 0.88 | 0.001 |  | 0.59 | 0.42 | - | 0.84 | 0.003 |
|  | ≥80 | | Ref. |  |  |  |  |  | Ref. |  |  |  |  |  | Ref. |  |  |  |  |
| Ethnicity | | |  |  |  |  | <0.001 |  |  |  |  |  | 0.004 |  | NA |  |  |  |  |
|  | Malay | | Ref. |  |  |  |  |  | Ref. |  |  |  |  |  |  |  |  |  |  |
|  | Chinese | | 1.00 | 0.93 | - | 1.06 | 0.894 |  | 0.84 | 0.72 | - | 0.99 | 0.032 |  |  |  |  |  |  |
|  | Indian | | 1.02 | 0.93 | - | 1.11 | 0.663 |  | 1.17 | 0.94 | - | 1.46 | 0.166 |  |  |  |  |  |  |
|  | Non-malay native | | 1.54 | 1.42 | - | 1.66 | <0.001 |  | 1.16 | 0.96 | - | 1.41 | 0.125 |  |  |  |  |  |  |
|  | Others | | 1.31 | 1.11 | - | 1.55 | 0.002 |  | 1.60 | 1.04 | - | 2.46 | 0.031 |  |  |  |  |  |  |
| Comorbidities: | | |  |  |  |  |  |  |  |  |  |  |  |  |  |  |  |  |  |
|  | Hypertension | |  |  |  |  | <0.001 |  | NA |  |  |  |  |  |  |  |  |  | 0.107 |
|  |  | No/unknown | Ref. |  |  |  |  |  |  |  |  |  |  |  | Ref. |  |  |  |  |
|  |  | Yes | 0.41 | 0.37 | - | 0.44 |  |  |  |  |  |  |  |  | 1.39 | 0.93 | - | 2.09 |  |
| DM complication: | | |  |  |  |  |  |  |  |  |  |  |  |  |  |  |  |  |  |
|  | Amputation | | NA |  |  |  |  |  |  |  |  |  | <0.001 |  |  |  |  |  | 0.080 |
|  |  | No/unknown |  |  |  |  |  |  | Ref. |  |  |  |  |  | Ref. |  |  |  |  |
|  |  | Yes |  |  |  |  |  |  | 2.19 | 1.44 | - | 3.32 |  |  | 2.28 | 0.91 | - | 5.76 |  |
| BMI Category,kgm^2^ | | |  |  |  |  | 0.958 |  |  |  |  |  | <0.001 |  | NA |  |  |  |  |
|  | 18.5-22.9 | | Ref. |  |  |  |  |  | Ref. |  |  |  |  |  |  |  |  |  |  |
|  | 23.0-27.4 | | 1.02 | 0.96 | - | 1.09 | 0.517 |  | 0.82 | 0.70 | - | 0.96 | 0.016 |  |  |  |  |  |  |
|  | 27.5-32.4 | | 1.02 | 0.95 | - | 1.09 | 0.585 |  | 0.75 | 0.64 | - | 0.89 | 0.001 |  |  |  |  |  |  |
|  | 32.5-37.4 | | 1.04 | 0.95 | - | 1.13 | 0.372 |  | 0.74 | 0.60 | - | 0.91 | 0.005 |  |  |  |  |  |  |
|  | ≥37.5 | | 1.05 | 0.93 | - | 1.17 | 0.436 |  | 0.60 | 0.45 | - | 0.81 | 0.001 |  |  |  |  |  |  |
|  | <18.5 | | 1.01 | 0.85 | - | 1.20 | 0.888 |  | 1.29 | 0.87 | - | 1.92 | 0.207 |  |  |  |  |  |  |
| Blood Pressure (mmHg) | | |  |  |  |  |  |  |  |  |  |  |  |  |  |  |  |  |  |
|  | Systolic: | | NA |  |  |  |  |  |  |  |  |  | 0.005 |  | NA |  |  |  |  |
|  |  | 120-130 |  |  |  |  |  |  | Ref. |  |  |  |  |  |  |  |  |  |  |
|  |  | >130 |  |  |  |  |  |  | 0.96 | 0.84 | - | 1.10 | 0.555 |  |  |  |  |  |  |
|  |  | <120 |  |  |  |  |  |  | 1.27 | 1.05 | - | 1.53 | 0.013 |  |  |  |  |  |  |
|  | Diastolic | |  |  |  |  | <0.001 |  |  |  |  |  | 0.202 |  | NA |  |  |  |  |
|  |  | 70-80 | Ref. |  |  |  |  |  | Ref. |  |  |  |  |  |  |  |  |  |  |
|  |  | >80 | 1.20 | 1.13 | - | 1.26 | <0.001 |  | 1.10 | 0.97 | - | 1.25 | 0.150 |  |  |  |  |  |  |
|  |  | <70 | 0.83 | 0.79 | - | 0.88 | <0.001 |  | 1.11 | 0.97 | - | 1.27 | 0.134 |  |  |  |  |  |  |
| Serum Cholesterol, mmol/L | | |  |  |  |  |  |  |  |  |  |  |  |  |  |  |  |  |  |
|  | Triglyceride | | NA |  |  |  |  |  |  |  |  |  | 0.673 |  | NA |  |  |  |  |
|  |  | ≤1.7 |  |  |  |  |  |  | Ref. |  |  |  |  |  |  |  |  |  |  |
|  |  | >1.7 |  |  |  |  |  |  | 0.98 | 0.87 | - | 1.09 |  |  |  |  |  |  |  |
|  | HDL^a^ | |  |  |  |  | <0.001 |  |  |  |  |  | 0.234 |  |  |  |  |  | 0.160 |
|  |  | Controlled | Ref. |  |  |  |  |  | Ref. |  |  |  |  |  | Ref. |  |  |  |  |
|  |  | Uncontrolled | 0.79 | 0.76 | - | 0.83 |  |  | 1.07 | 0.96 | - | 1.20 |  |  | 1.13 | 0.95 | - | 1.34 |  |
|  | LDL | |  |  |  |  | <0.001 |  |  |  |  |  | 0.039 |  |  |  |  |  | <0.001 |
|  |  | ≤2.6 | Ref. |  |  |  |  |  | Ref. |  |  |  |  |  | Ref. |  |  |  |  |
|  |  | >2.6 | 1.08 | 1.04 | - | 1.13 |  |  | 1.12 | 1.01 | - | 1.25 |  |  | 1.56 | 1.32 | - | 1.85 |  |

Footnotes: AOR, adjusted odds ratio; CI, confidence interval; NA, not applicable (excluded from the final multivariable model because of significant interaction); Ref., reference; IHD, ischaemic heart disease; CeVD, cerebrovascular disease; DFU, diabetic foot ulcer; BMI, body mass index; HbA1c, glycosylated haemoglobin A1c; HDL, high-density lipoprotein; LDL, low-density lipoprotein

^a^Definition of controlled HDL levels for male: >1·0 mmol/L, female: >1·2 mmol/L was based on the Clinical Practice Guidelines Management of Type 2 Diabetes Mellitus (6^th^ Edition) recommendations
