# Supplementary material for: Navigating the kidney disease care cascade in type 2 diabetes: insights from the national diabetes registry
Source: BMC Public Health. 2026 Jul 27;26:3035. doi: 10.1186/s12889-026-28520-9 (PMC13689289; doi:10.1186/s12889-026-28520-9)
Supplement: Supplementary file 2 [file 12889_2026_28520_MOESM2_ESM.docx]

| **Supplementary Table 2**: Descriptive distributions of the DKD care cascade across disease stages. | | | | | |  |
| --- | --- | --- | --- | --- | --- | --- |
| DKD Stage | Diagnosis Stage (n=56949) | | Treatment Stage (n=8394) | | Control Stage (n=5998) | |
|  | Diagnosed (n=14437) | Undiagnosed (n=42512) | Treated (n=6053) | Untreated (n=2341) | Controlled (n=793) | Uncontrolled (n=5205) |
| 1 | 2332 (16.2) | 13273 (31.2) | 1032 (17.0) | 393 (16.8) | 94 (11.9) | 931 (17.9) |
| 2 | 3917 (27.1) | 13777 (32.4) | 1792 (29.6) | 559 (23.9) | 260 (32.8) | 1527 (29.3) |
| 3 | 5361 (37.1) | 12652 (29.8) | 2424 (40.1) | 844 (36.0) | 357 (45.0) | 2051 (39.4) |
| 4 | 1277 (8.9) | 1359 (3.2) | 548 (9.1) | 316 (13.5) | 64 (8.0) | 478 (9.2) |
| 5 | 1550 (10.7) | 1451 (3.4) | 257 (4.2) | 229 (9.8) | 18 (2.3) | 218 (4.2) |
| Total | 14437 (100) | 42512 (100) | 6053 (100) | 2341 (100) | 793 (100) | 5205 (100) |
| Note: Data are presented as n (percentage). Percentages indicate the proportion of patients within each step of the DKD care cascade. | | | | | | |
